# Genetic manipulation of pathogenic *Leptospira*: CRISPR interference (CRISPRi)-mediated gene silencing and rapid mutant recovery at 37 °C

**DOI:** 10.1038/s41598-021-81400-7

**Published:** 2021-01-19

**Authors:** L. G. V. Fernandes, R. L. Hornsby, A. L. T. O. Nascimento, J. E. Nally

**Affiliations:** 1 Infectious Bacterial Diseases Research Unit, National Animal Disease Center, Agricultural Research Service, United States Department of Agriculture, Ames, IA USA; 2 Laboratório de Desenvolvimento de Vacinas, Instituto Butantan, São Paulo, 05503-900 Brazil

**Keywords:** Genetic engineering, Genetic techniques, Microbiology techniques

## Abstract

Leptospirosis is a neglected, widespread zoonosis caused by pathogenic species of the genus *Leptospira,* and is responsible for 60,000 deaths per year. Pathogenic mechanisms of leptospirosis remain poorly understood mainly because targeted mutations or gene silencing in pathogenic *Leptospira* continues to be inherently inefficient, laborious, costly and difficult to implement. In addition, pathogenic leptospires are highly fastidious and the selection of mutants on solid agar media can take up to 6 weeks. The catalytically inactive Cas9 (dCas9) is an RNA-guided DNA-binding protein from the *Streptococcus pyogenes* CRISPR/Cas system and can be used for gene silencing, in a strategy termed CRISPR interference (CRISPRi). Here, this technique was employed to silence genes encoding major outer membrane proteins of pathogenic *L. interrogans*. Conjugation protocols were optimized using the newly described HAN media modified for rapid mutant recovery at 37 °C in 3% CO_2_ within 8 days. Complete silencing of LipL32 and concomitant and complete silencing of both LigA and LigB outer membrane proteins were achieved, revealing for the first time that Lig proteins are involved in pathogenic *Leptospira* serum resistance. Gene silencing in pathogenic leptospires and rapid mutant recovery will facilitate novel studies to further evaluate and understand pathogenic mechanisms of leptospirosis.

## Introduction

Leptospirosis is a neglected, widespread zoonosis caused by distinct pathogenic species of the genus *Leptospira*, and is responsible for more than one million cases and 60,000 deaths per year worldwide^1–3^. Domestic and wild animal populations can be asymptomatic carriers of the pathogen, which are excreted from persistently colonized renal tubules via urine into the environment to maintain continued disease transmission^1,4^. Humans and other animals are infected via contact with urine of infected hosts, either directly, or indirectly through contaminated water or soil^3^. Humans are considered an incidental and terminal host, exhibiting a wide variety of symptoms, ranging from nonspecific symptoms such as fever, chills, headache and myalgia, to severe leptospirosis, a condition known as Weil's disease^1,5^.

Understanding pathogenic mechanisms of leptospirosis, and the identification of virulence factors, is a critical step in the development of better therapeutic and prophylactic strategies. In this context, functional genomic analysis requires an ability to generate genetic mutations and assess the resulting phenotype^6^. Despite the recent advances for genetic manipulation of many microbial species, targeted mutations or gene silencing in pathogenic *Leptospira* continues to be inherently inefficient, laborious, costly and difficult to implement. Gene disruption in pathogenic leptospires relies on random transposon insertion or targeted genetic knockout by infrequent homologous recombination events, coupled with weeks waiting for the growth and selection of colonies on agar media^7–9^.

To overcome the double recombination requirement for allelic exchange, episomal targeted gene silencing techniques are emerging as an alternative and efficient approach for assessing gene function; the prokaryote immunity type II CRISPR/Cas (clustered regularly interspaced short palindromic repeat/CRISPR associated) from *Streptococcus pyogenes* has been widely used as an efficient biotechnological tool for genome engineering in various organisms due to its two-component simplicity^10,11^. The Cas9 enzyme is a DNA endonuclease that can be programmed to target genomic sites depending on the interaction with the single-guide RNA sequence (sgRNA)^12^, which dictates the target for the endonuclease Cas9, by Watson and Crick base pairing. Cas9 recognition of a protospacer adjacent motif (PAM) and subsequent RNA–DNA base pairing lead to DNA cleavage, provoking double-strand breaks (DSB) that need to be repaired for cell viability^13,14^. DSB in the genome of most bacteria is reported to be lethal in the absence of a template for recombination^14,15^. Cas9-induced DSB lethality in the saprophyte *L. biflexa* has previously been demonstrated^16^. A variant of Cas9 lacking nuclease activity, called catalytically dead Cas9 (dCas9), has been extensively employed to overcome this barrier. In this case, RNA-guided dCas9 protein is capable of binding specific DNA targets, causing a steric hindrance to RNA polymerase, resulting in gene silencing rather than disruption due to blockage of gene transcription^17^. This strategy, called CRISPRi, has been successfully applied in various organisms^18–22^, including the saprophyte *L. biflexa*^16^, in which specific and stable gene silencing was observed for different targets.

In this work, we report for the first time the successful application of CRISPRi to pathogenic species of *Leptospira*, using an optimized protocol for conjugation and the selection of transconjugants using the recently described Hornsby-Alt-Nally (HAN) media^23^. Recombinant pathogenic leptospiral colonies could be recovered as soon as 8 days in solid media at 37 °C and complete and stable silencing of genes encoding outer membrane proteins was demonstrated.

## Materials and methods

### Bacterial strains and media

Pathogenic *L. interrogans* serovar Copenhageni strain FIOCRUZ L1-130 and a recently isolated pathogenic non-*interrogans Leptospira* strain LGVF02 from soil samples (Nathan Stone, Northern Arizona University, manuscript in preparation) were grown in liquid HAN media^23^ at 29 °C. Saprophytic *L. biflexa* serovar Patoc strain Patoc1 was cultured in EMJH medium (Difco, BD, Franklin Lakes, NJ). Solid media were prepared by supplementing with 1.2% noble agar (Difco). Where necessary, spectinomycin was added at 40 µg/mL. *E. coli* strains π1 and β2163^24^ were used for general cloning and as conjugation donor cells, respectively, and were grown in Luria–Bertani (LB, Difco) media. For *E. coli* strains π1, thymidine (dT, 0.3 mM, Sigma) was added to the media and for strain β2163, diaminopimelic acid (DAP, 0.3 mM, Sigma) was added.

### Plasmids and sgRNA construction

**Figure 1:**
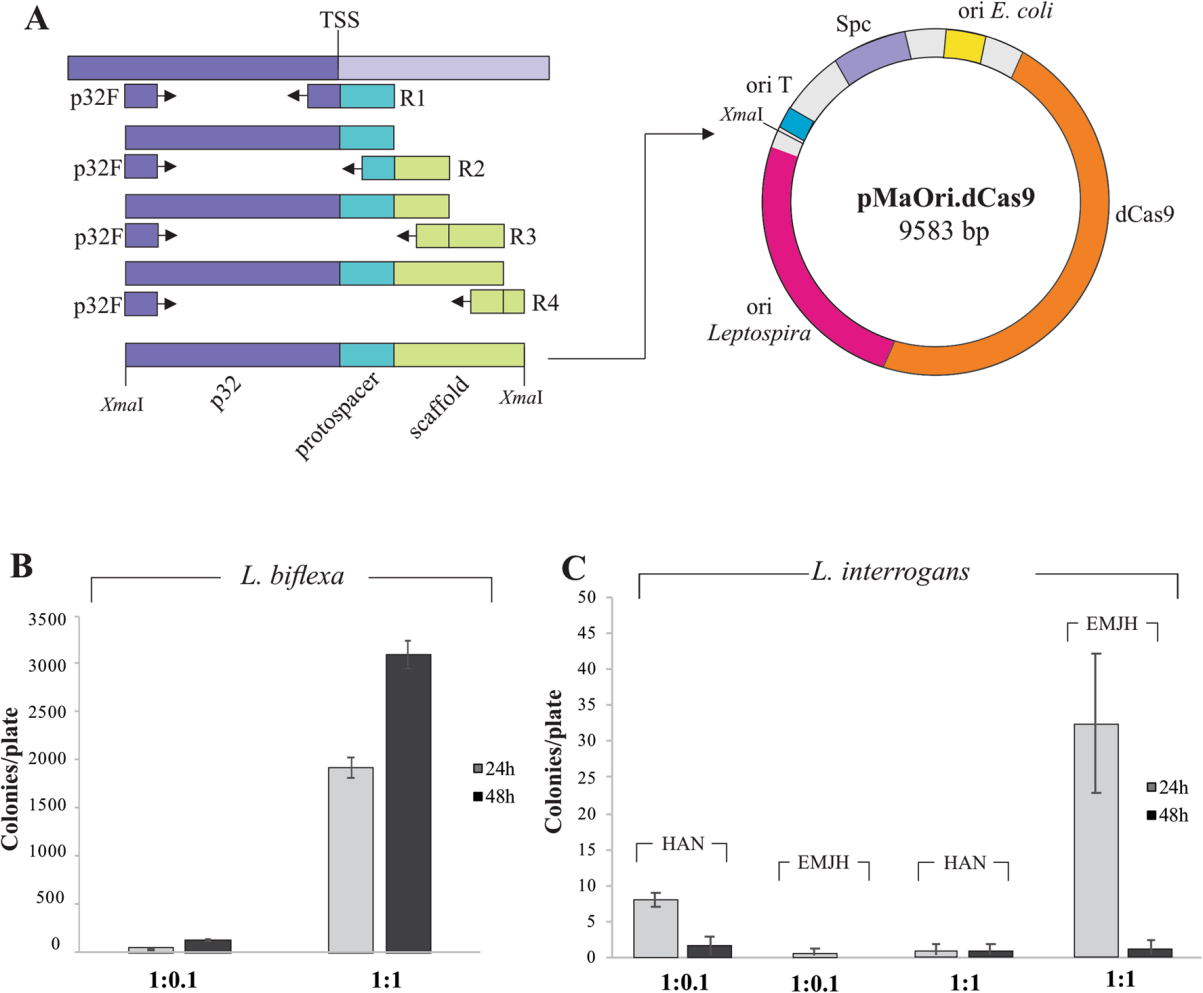
Plasmid construction and optimization of conjugative transfer. (**A**) sgRNA cassettes were obtained by sequential PCR starting with the *lipL32* promoter amplified from the genome of *L. interrogans*, and ligated into pMaOri.dCas9 plasmid at *Xma*I. Plasmid pMaOri.dCas9sg32 (targeting *lipL32* CDS) was used to transform the conjugative *E. coli* β2163, which was concentrated by filtration with recipient wild type *L. biflexa* (**B**) or *L. interrogans* (**C**). The recipient:donor proportions of 1:0.1 and 1:1 were employed and filter mating proceeded by either 24 or 48 h. For *L. interrogans* conjugation, filters were placed onto EMJH or HAN plates plus DAP. Results refer to the average ± SD of recovered colonies after 2 experiments for *L. biflexa* and 4 experiments for *L. interrogans*.

The plasmid pMaOri.dCas9, as previously developed for gene silencing in the saprophyte *L. biflexa*^16^, was employed for sgRNA cassette ligation. The cassette comprised the constitutive *lipL32* promoter from *L. interrogans* (334 nucleotides upstream of TSS, as defined by Zhukova et al*.*^25^), a 20 bp protospacer specific for the selected gene target, and a *S. pyogenes* dCas9 scaffold. Protospacer sequences were designed according to proximity to the 5′ end of selected genes, strand localization, score, and protospacer adjacent motif 5′-NGG-3′, by the webserver CHOPCHOP (https://chopchop.cbu.uib.no)^26^. Selected protospacer and gene targets are displayed in Table 1, and Supplementary Fig. 1. In order to synthesize the final sgRNA cassette specific for *lipL32*, the *lipL32* promoter was first amplified by PCR from *L. interrogans* serovar Copenhageni strain FIOCRUZ L1-130 genomic DNA with primers p32F and sgLipL32 R1, Fig. 1A and Table 2. The resulting amplicon was used as template for PCR with p32F and sgLipL32 R2. Sequential PCRs were further performed with p32F and reverse primer R3, and p32F and reverse primer R4, to complete synthesis of the final cassette as schematized in Fig. 1A. The sgRNA cassette specific for *ligA* and *ligB* was synthesized in a similar manner except that reverse primers sgLigAB R1 and sgLigAB R2 replaced primers sgLipL32 R1 and sgLipL32 R2, respectively, in each PCR reaction. Final amplicons were digested with *Xma*I and ligated into pMaOri.dCas9 previously digested with the same enzyme in a T4 DNA ligase-mediated reaction at the insert:plasmid molar ratio of 10:1. Ligation reactions were used to transform *E. coli* π1 cells, which were plated onto LB solid media supplemented with dT (0.3 mM) and spectinomycin (40 μg/mL). Clones were screened by colony PCR with primers pMaOri2 F and R and then grown in liquid media for plasmid extraction by *illustra plasmidPrep Mini Spin Kit* (GE). Recombinant plasmids were confirmed by restriction enzyme analysis and sequencing.

**Table 1:** Plasmids and protospacers composing the sgRNA and specificity.

Plasmids	Protospacer	Target
pMaOri.dCas9	–	–
pMaOri.dCas9sg32	5′-ACCACCGAAAGCACCACAAG-3′	*lipL32*
pMaOri.dCas9sgLigAB	5′-ACCATCCGAAAAGATACCGA-3′	*ligA* and *ligB*
pMaOri.dCas9sgLigBMismatch	5′-CGTTCCATCATCAAAGATCG-3′	–^a^

^a^Designed for *L. borgpetersenii* ligB.

**Table 2:** Primers.

Primer name	Sequence (5′ → 3′)	Restriction site
P32 F	ATATCCCGGGGAACAAGAAAGAGTCAGAG	*Xma*I
sgLipL32 R1	TAGCTCTAAAACCTTGTGGTGCTTTCGGTGGTGAAAATCACGGTATGAAC	–
sgLipL32 R2	TAACGGACTAGCCTTATTTTAACTTGCTATTTCTAGCTCTAAAACCTTGT	–
sgLigAB R1	TAGCTCTAAAACTCGGTATCTTTTCGGATGGTGAAAATCACGGTATGAAC	–
sgLigAB R2	AACGGACTAGCCTTATTTTAACTTGCTATTTCTAGCTCTAAAACTCGGTA	–
sgLigBMismatch R1	TAGCTCTAAAACCGATCTTTGATGATGGAACGGAAAATCACGGTATGAAC	
sgLigBMismatch R2	AACGGACTAGCCTTATTTTAACTTGCTATTTCTAGCTCTAAAACCGATCT	–
R3	CCACTTTTTCAAGTTGATAACGGACTAGCCTTATTTTAAC	–
R4	ATACCCGGGAAAAAAGCACCGACTCGGTGCCACTTTTTCAAGTTGATAAC	*Xma*I
pMaOri2 F	ACGCAATGTATCGATACCGAC	–
pMaOri2 R	ATAGGTGAAGTAGGCCCACCC	–
qLipL32 F	GCATTACCGCTTGTGGTG	–
qLipL32 R	CCGATTTCGCCTGTTGG	–
qSecY F	GCGATTCAGTTTAATCCTGC	–
qSecY R	GAGTTAGAGCTCAAATCTAAG	–
Spc F	CGAATGTGGCAAACAGTGAC	–
Spc R	AACGAGTGCTTTCACCTTTGA	–

Underlined nucleotides denote restriction sites.

### Transformation of *Leptospira*

Transformation protocols were assessed with different recipient and donor cell proportions (1:0.1 or 1:1), conjugation times (24 or 48 h) and media (EMJH or HAN). The donor *E. coli* β2163 strain was transformed with plasmids by electroporation (1.8 kV, 25 μF and 200 Ω), plated onto LB solid media containing DAP (0.3 mM) and spectinomycin (40 μg/mL) and a single colony was picked for overnight growth in liquid LB with the same supplementation. Then, saturated cultures were re-diluted in LB plus DAP and incubated until OD_420nm_ of 0.3–0.5 for conjugation with leptospiral strains, previously grown to OD_420nm_ of 0.3–0.5 (mid-log phase). Five mL of recipient leptospiral suspensions were mixed with either 0.5 or 5 mL of recombinant donor *E. coli* by filtration on the surface of a 0.1 μm pore size 25 mm mixed cellulose esters hydrophilic membrane (Millipore Sigma). Filters were transferred to either EMJH or HAN agar plates containing DAP and incubated for 24 or 48 h at 29 °C. After conjugation, *L*. *interrogans* was subsequently recovered by continuous pipetting of 1 mL of HAN media to the filters, as confirmed by dark field microscopy, and then 100 μL seeded onto HAN agar plates containing 0.4% inactivated rabbit serum (56 °C, 20 min) and spectinomycin (40 μg/mL) and incubated at 29, or 37 °C with 3% CO_2_; at this stage, DAP is omitted. Colonies were recovered and vigorously homogenized in 100 μL HAN media to release leptospires from the agar, and suspensions were visualized by dark field microscopy to confirm the presence of motile leptospires. Finally, 50 μL of each homogenate were inoculated into 5 mL liquid HAN plus spectinomycin. After visible growth, cultures were assessed by PCR with pMaOri2 F and R primers to confirm the presence of plasmids and sgRNA cassettes.

### Scanning electron microscopy

Procedures and sample preparation were performed as described^27,28^. In brief, recovered cells after 24 h conjugation were diluted 100X in HAN media and put through a 0.22 μm Swinney filter (Millipore), which was fixed in 2.5% glutaraldehyde in 0.1 M cacodylate buffer. The samples were stained by sequential exposure to osmium and thiocarbohydrazide (OTOTO). Samples were dehydrated through graded alcohols and chemically dried with hexamethyldisilizane (HMDS). Samples were coated with a gold/palladium mixture and viewed on the Hitachi TM3030Plus SEM.

### Fluorescent antibody test

After colony recovery from agar plates, 10 μL of the homogenized suspensions were placed on a glass slide with a 7 mm well for the fluorescent antibody test (FAT), as previously described^29^. In brief, slides were air dried overnight and fixed in acetone for 15 min. Slides were then placed in a humid chamber and 50 μL of rabbit anti-LipL32 sera (1:250) was applied to each 7 mm spot. Slides were incubated at 37 °C for 1 h and subsequently washed for 10 min in PBS with gentle rocking. Next, slides were incubated with secondary Alexa Fluor 488 F(ab’)2 goat anti-rabbit IgG (Invitrogen) for 1 h at 37 °C in the dark. After extensive washing, slides were dried and counterstained for 15 s with Flazo Orange (1:50, National Veterinary Services Laboratory). Slides were rinsed with PBS and mounted using ProLong Diamond Antifade Mountant with DAPI (Thermo-Fisher Scientific). Microscopic examination was done using a Nikon Eclipse E800 microscope and B2-A filter (excitation, 450–490 nm; emission, 520 nm) at 400 × magnification.

### Electrophoresis and immunoblotting

Cultures of *Leptospira* at mid to late-log phase of growth were centrifuged (10,000×*g*, 30 min, 4 °C) and the resulting pellet was washed twice with sterile PBS. Protein samples were processed for one-dimensional (1-D) SDS-PAGE on 12% acrylamide gels (BioRad) as per manufacturer’s guidelines. Proteins were visualized by staining with Sypro Ruby (Invitrogen, CA) as per manufacturer’s guidelines. For immunoblotting, proteins were electrotransferred onto polyvinylidene difluoride (PVDF) membranes (BioRad), previously activated by methanol, by semidry transfer. Membranes were blocked with SuperBlock (PBS) Blocking Buffer (Thermo) for 1 h and then incubated with indicated primary antibody diluted in the blocking buffer (1:4000) for 1 h at room temperature. Membranes were then washed 3 times with PBS 0.1% Tween 20 (PBS-T) and incubated with horseradish peroxidase-conjugated secondary anti-rabbit IgG (1:4000) antibody in blocking buffer for 1 h at room temperature. After extensive washing, detection of antigen reactivity was revealed using Clarity and Clarity Max ECL (BioRad) and the luminescence generated was detected with a ChemiDoc MP Imaging System (BioRad).

### RNA extraction and quantitative RT-PCR

*Leptospira* cells in 5 mL HAN medium were collected by centrifugation (12,000×*g* for 15 min at 4 °C) in RNase-free tubes. Bacterial pellets were resuspended in 1 mL of Trizol reagent (Invitrogen), and total RNA was purified according to the methods described by Zavala-Alvarado et al*.*^30^, followed by incubation with Turbo DNase (ThermoFisher) to eliminate residual DNA. Sample quality and concentration were assessed by Qubit Fluorometer (Thermofisher Scientific, CA, USA) as per manufacturer instructions. cDNA was synthesized using 1 µg total RNA, the iScript cDNA synthesis Kit (Biorad), according to standard protocols. A control reaction where reverse transcriptase was omitted was also employed (RT negative control). qPCR was performed using the CFX384 Real-Time System (Bio-Rad), using SYBR Green I dye and primers as listed in Table 2. Reactions were performed in triplicate in a volume of 20 μL each containing 1 µL of cDNA, 400 nM of each oligonucleotide and 10 μL of SYBR Green PCR Master Mix (Applied Biosystems), as recommended by the manufacturer. Negative controls using all the reagents except cDNA (NTC, no template control) were also included. Cycling conditions were: 50 °C for 2 min and 95 °C for 10 min, followed by 40 cycles of 95 °C for 15 s and 60 °C for 30 s. The relative gene expression among wild type and mutant cells containing different plasmids was determined using the 2^–ΔΔCT^ method. The constitutively expressed *secY* gene^31^ was used as the internal normalization control.

### Plasmid and phenotype stability

*Leptospira interrogans* harboring the pMaOri.dCas9sg32 plasmid was grown in HAN containing spectinomycin and then transferred to new HAN media with no antibiotic at the initial inoculum of 10^6^ cells/mL. Serial passages were performed in HAN media and at each passage, cells were collected for whole cell extract preparations as described above, which were evaluated by immunoblotting. Total DNA at each passage was also extracted by QIAamp DNA Mini for quantitative PCR, as described above, with primers Spc F and R (targeting the spectinomycin resistance gene in the pMaOri backbone) and *lipL32* primers as an internal control (Table 2).

### Serum resistance assay

*L. interrogans* containing the plasmids pMaOri.dCas9 alone or with the sgRNA targeting *ligA* and *ligB* were brought to 10^5^ cells/mL in PBS containing 25% bovine serum (Sigma), heat inactivated (56 °C, 20 min) or not, and incubated for 24 h at 37 °C. Different dilutions of the suspensions were plated onto HAN plates and incubated at 37 °C and 3% CO_2_. The serum bactericidal effect on each population of leptospires was assessed by colony counting and significance determined using the two-tailed Student's t-test. The number of recovered colonies after heat-inactivated serum treatment within each group was considered as 100% of survival.

## Results

### Optimal conjugation and rapid transconjugant recovery

Protospacers within the template strand of target genes were selected for sgRNA pairing to the coding strand, a requirement for full gene silencing in *Leptospira* as well as other bacteria^16,18,19,21,22^. Selected 20 bp protospacers and target genes are listed in Table 1. sgRNA cassettes, comprising *lipL32* promoter, protospacer and *S. pyogenes* dCas9 scaffold, were successfully cloned into the pMaOri.dCas9 plasmid, Fig. 1A. dCas9 transcription is driven by the native *S. pyogenes cas9* promoter; though previously demonstrated to be fully functional in the saprophytic species *L. biflexa*, its functionality in pathogenic species remained to be determined.

CRISPRi plasmids for gene silencing in the saprophyte *L. biflexa* were previously delivered by electroporation, and bacteria were recovered and plated using EMJH growth media^16^. However, given the more fastidious nature of pathogenic species compared to saprophytic species of leptospires, and the limited efficiencies of electroporation, plasmid delivery was optimized using conjugation and plating in the newly described HAN media. This media supports the growth of more fastidious species/serovars of pathogenic leptospires compared to EMJH, and facilitates growth at both 29 and 37 °C^23^.

Initial variables tested for optimal conjugation in *L*. *biflexa* included recipient:donor cell proportions, and conjugation incubation times of 24 or 48hrs. Recipient *L. biflexa* cells were mixed with the donor recombinant β2163 *E. coli* containing the plasmid pMaOri.dCas9sg32 by filtration, at a recipient:donor cell proportion of 1:0.1 and 1:1; filters were placed onto EMJH agar plates plus DAP and conjugation proceeded for 24 or 48 h. After transconjugants were recovered from filters and plated on EMJH agar plates, higher efficiencies of conjugation were observed with a reciprocal cell proportion of 1:1, with increased transconjugants recovered using a 48 h conjugation time, Fig. 1B. This optimal 1:1 proportion for filter mating was also observed by Lampkowska et al.^32^ for lactococcal species.

To optimize conjugation and recovery in pathogenic species, variables tested included recipient:donor cell proportions, conjugation incubation times of 24 or 48hrs, and using EMJH or HAN solid media for filter mating. Among the combinations tested, optimal recovery of transconjugants for *L. interrogans* serovar Copenhageni strain FIOCRUZ L1-130 occurred using mating on EMJH plus DAP at a donor:recipient proportion of 1:1 for 24 h, Fig. 1C. Recombinant colonies were also recovered using a conjugation incubation time of 24 h in HAN at a donor:recipient proportion of 1:0.1. In all cases, transconjugants were recovered from filters by plating on HAN agar media containing 0.4% rabbit serum plus spectinomycin at 37 °C. Transconjugant recovery typically took 8–10 days in these conditions; in control plates with no antibiotic, colonies could be observed in 7 days. Incubation at 29 °C rendered no visible colonies in up to 30 days of culture. HAN agar plates supported the relatively rapid growth of pathogenic leptospires after conjugation in 8–10 days, but did not effectively facilitate the conjugation process; on average, only 8 transconjugants per plate were recovered after conjugation for 24 h in HAN compared to 32 transconjugants recovered after conjugation in EMJH, Fig. 1C.

Visualization of pathogenic *Leptospira* and *E*. *coli* recovered from filters after conjugation for 24 h in EMJH or HAN media indicated that the initial donor:recipient proportions of 1:0.1 and 1:1 were only preserved when mating occurred on the surface of EMJH plates, Supplementary Fig. 2A. The recipient:donor proportion of 1:1 for conjugation using EMJH is similar to that of HAN using a recipient:donor proportion of 1:0.1. This suggests that *E. coli* proliferates in HAN compared to EMJH and as confirmed by growth curves of wild type β2163 *E. coli* in EMJH and HAN supplemented with DAP, Supplementary Fig. 2B,C respectively. Such proliferation alters the intended donor:recipient proportions when conjugation occurs in HAN plates; It was previously shown that an excess of donor cells impairs conjugation efficiency^32^. Accordingly, all future conjugations with pathogenic leptospires were performed by filter mating in EMJH plus DAP plates for 24 h with a 1:1 proportion and suspensions were seeded onto HAN agar plates plus 0.4% rabbit serum and incubated at 37 °C in a 3% CO_2_ atmosphere. Scanning electron microscopy confirmed the close association of *E. coli* to *Leptospira,* denoting conjugation events, as presented in Supplementary Fig. 3.

**Figure 2:**
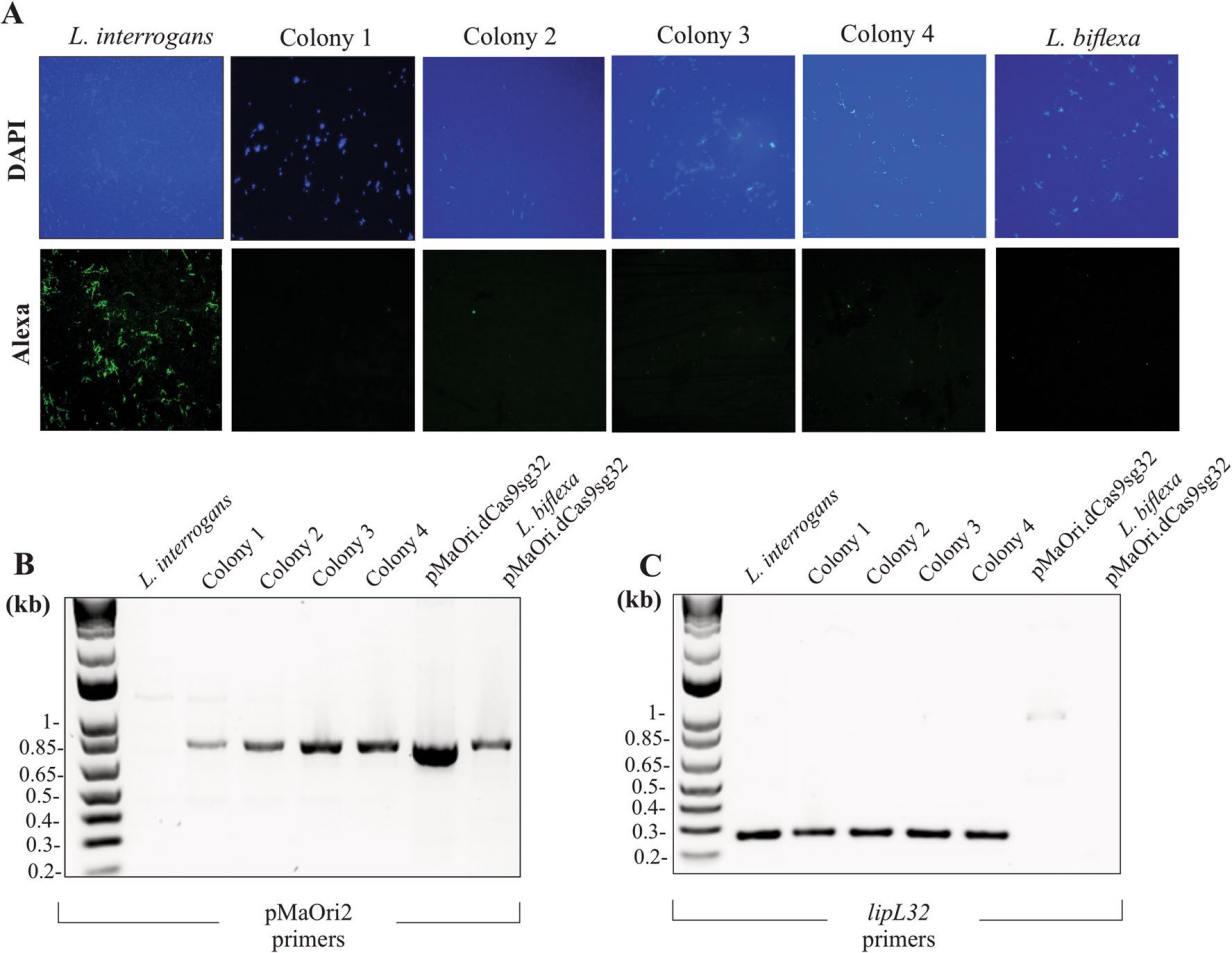
Initial assessment of gene silencing and plasmid detection. Different colonies of *L. interrogans* serovar Copenhageni strain FIOCRUZ L1-130 transformed with plasmid pMaOri.dCas9sg32 were picked from HAN plates, homogenized in liquid HAN and were placed on a glass slide for FAT with anti-LipL32 antibodies (**A**). *Leptospira* cells can be visualized with DAPI staining, and specific reactivity to LipL32 with Alexa fluorophore. Cells were grown in liquid HAN plus spectinomycin and then evaluated by PCR for the presence of the plasmids with primers flanking the sgRNA cassette (**B**) or *lipL32* (**C**). Plasmid pMaOri.dCas9sg32 or *L. biflexa* containing this plasmid were also employed as templates.

**Figure 3:**
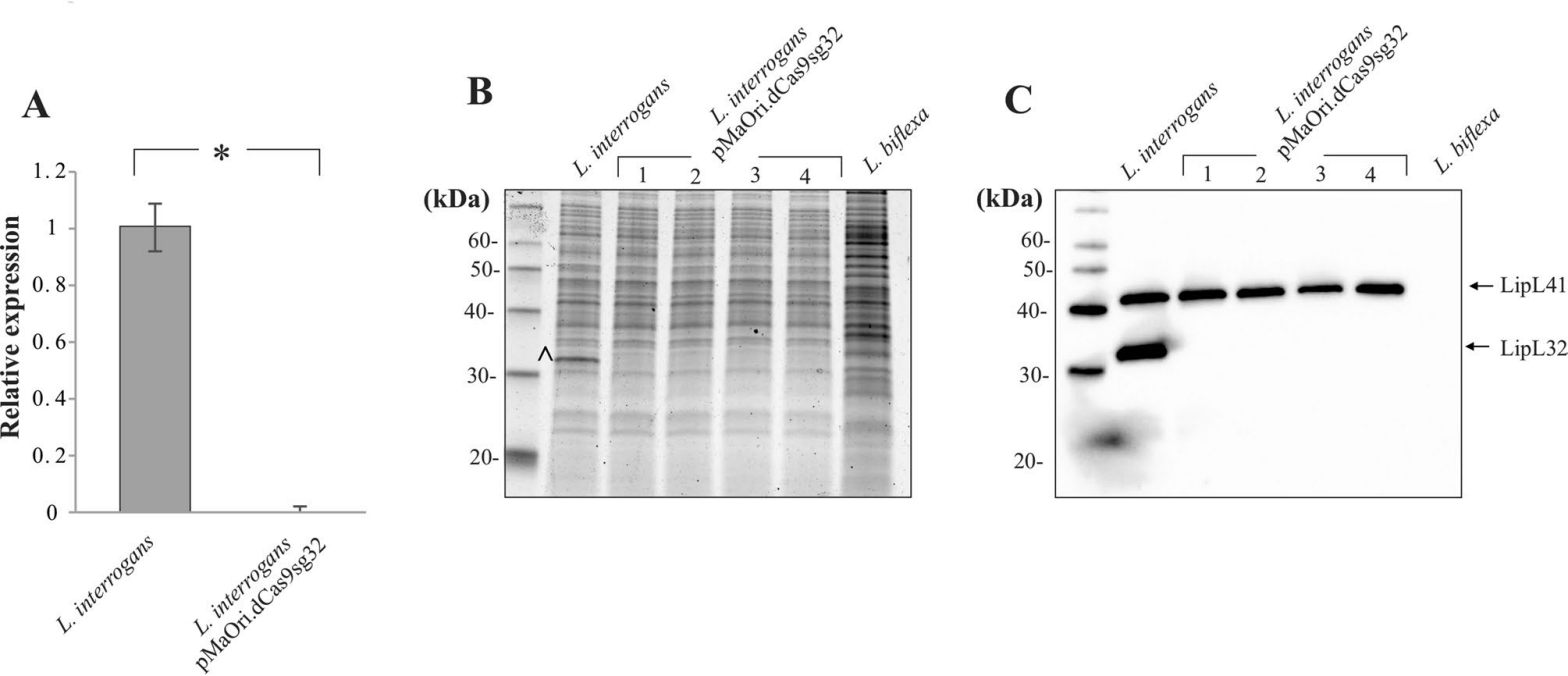
Silencing of LipL32. mRNA levels of *lipL32* were measured indirectly by cDNA synthesis from RNA isolated from wild type *L. interrogans* or a pool of recombinant cells containing the plasmid pMaOri.dCas9sg32 by qPCR (**A**). Cellular extracts of wild type *L. interrogans* and *L. biflexa*, and of recombinant cells derived from 4 different colonies were evaluated by SDS-PAGE (**B**) and immunoblotting (**C**), with antibodies specific for LipL32 and LipL41. ^indicates a protein with an apparent molecular mass of 32 kDa. Molecular mass markers (kDa) are indicated. SE bars represent standard errors, and *denotes significance of *p* value < 0.05.

### *lipL32* gene silencing in *L. interrogans*

As proof of concept, the gene encoding LipL32, the major outer membrane protein conserved among pathogenic *Leptospira* species^33^, was selected for silencing. Protein dCas9, guided by a sgRNA specific for the coding strand of the *lipL32* gene, should cause a steric hindrance upon RNA polymerase elongation, and thus reducing, or abolishing mRNA levels. Transconjugant colonies selected from agar plates were homogenized in HAN media; 10 μl was used for FAT and the remainder for propagation in liquid HAN growth media plus spectinomycin, Fig. 2A. The immediate FAT analysis of cells directly recovered from colonies provides for rapid confirmation of the success of gene silencing. After incubation with polyclonal rabbit anti-LipL32 and Alexa-conjugated secondary antibodies, strong LipL32 reactivity (green) concordant with leptospiral cell morphology could be observed in control *L. interrogans* recovered from HAN plates with no antibiotic. In contrast, no apparent reactivity was observed in the cells transformed with pMaOri.dCas9sg32, or in *L. biflexa* cells (negative control), indicating that recombinant cells have reduced or abolished LipL32 protein production (Fig. 2A). After growth in liquid HAN plus spectinomycin, cells were evaluated for the presence of plasmid and *lipL32* by PCR (Fig. 2B,C); results demonstrate that all colonies assayed were positive for the presence of the plasmid.

Transcript levels of *lipL32* were evaluated by RT-qPCR in both wild type and pooled recombinant cells confirming that gene transcription is silenced by the RNA-guided dCas9, Fig. 3A. Next, LipL32 protein levels were evaluated in the recombinant cells. As shown in Fig. 3B, a band with an apparent molecular mass of 32 kDa can be observed in wild type *L. interrogans*, but is absent in *L. biflexa* and in *L. interrogans* containing pMaOri.dCas9sg32. Total protein profiles were evaluated by immunoblotting with anti-LipL32 and anti-LipL41 antibodies and demonstrate that no LipL32 protein could be detected in recombinant cells, even with longer exposure times (data not shown), confirming the applicability of CRISPRi to this strain, Fig. 3C.

### Evaluation of phenotype stability

**Figure 4:**
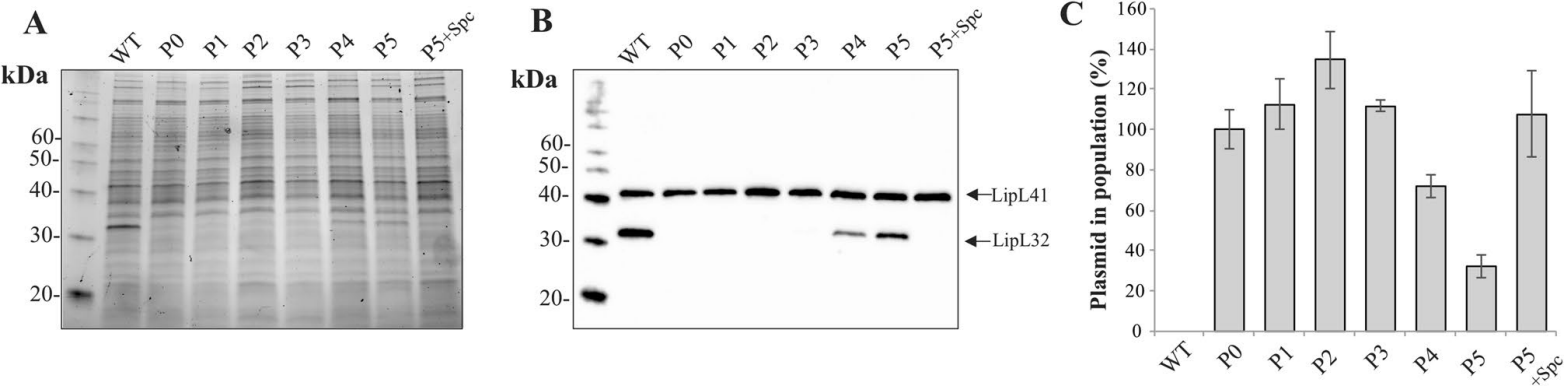
Evaluation of phenotypic stability of gene silencing. Protein extracts from wild type *L*. *interrogans* (WT) or recombinant cells containing pMaOri.dCas9sg32 were evaluated by SDS-PAGE (**A**) and immunoblotting (**B**). Recombinant cells were grown in the presence of spectinomycin, P0, and then serially grown in liquid HAN with no antibiotic pressure selection (P1–P5). Addition of spectinomycin at passage 5 (P5 + Spc) fully restored gene silencing. Genomic DNA of cells at each passage was evaluated by qPCR with primers specific for the pMaOri backbone and the *lipL32* primer set was employed as an internal control (**C**). Results are expressed as a % of plasmid in the total population, where P0 was considered 100%.

Considering that CRISPRi is an episomal tool of gene silencing rather than gene disruption, the continued silencing of *lipL32* was evaluated in cells after multiple passages in HAN growth media with no antibiotic selective pressure. Cells at passage 0 (grown in spectinomycin) and passage numbers 1 to 5 (without antibiotic) were evaluated for expression of LipL32 by protein gels, as visualized by Sypro Ruby protein stain, and immunoblotting with specific antisera, Fig. 4A,B respectively, as well as by qPCR to detect the presence of the plasmid, Fig. 4C.

Robust and stable silencing of LipL32 could be observed up to passage number 3 in transconjugants, in agreement with plasmid detection in those cells. These results indicate that recombinant cells are suitable for downstream experimental analysis in the absence of antibiotic selective pressure. At passage numbers 4 and 5, a modest and continued increase in expression of LipL32 protein could be observed, though it was still at lower levels when compared to that in the wild type strain, and concordant with a decrease in detection of the plasmid in the population. Complete gene silencing could be restored when transconjugants were re-grown in HAN plus spectinomycin (P5 + Spc), indicating that there were still plasmid-harboring leptospires in the cultures. The number of plasmids per cell is approximately 1, as determined by qPCR of cells from passage number 0, and comparing *Ct* values displayed by *lipL32* and pMaOri amplifications curves (data not shown).

### LigA and LigB silencing in *L. interrogans* decreases bacterial survival upon serum challenge

**Figure 5:**
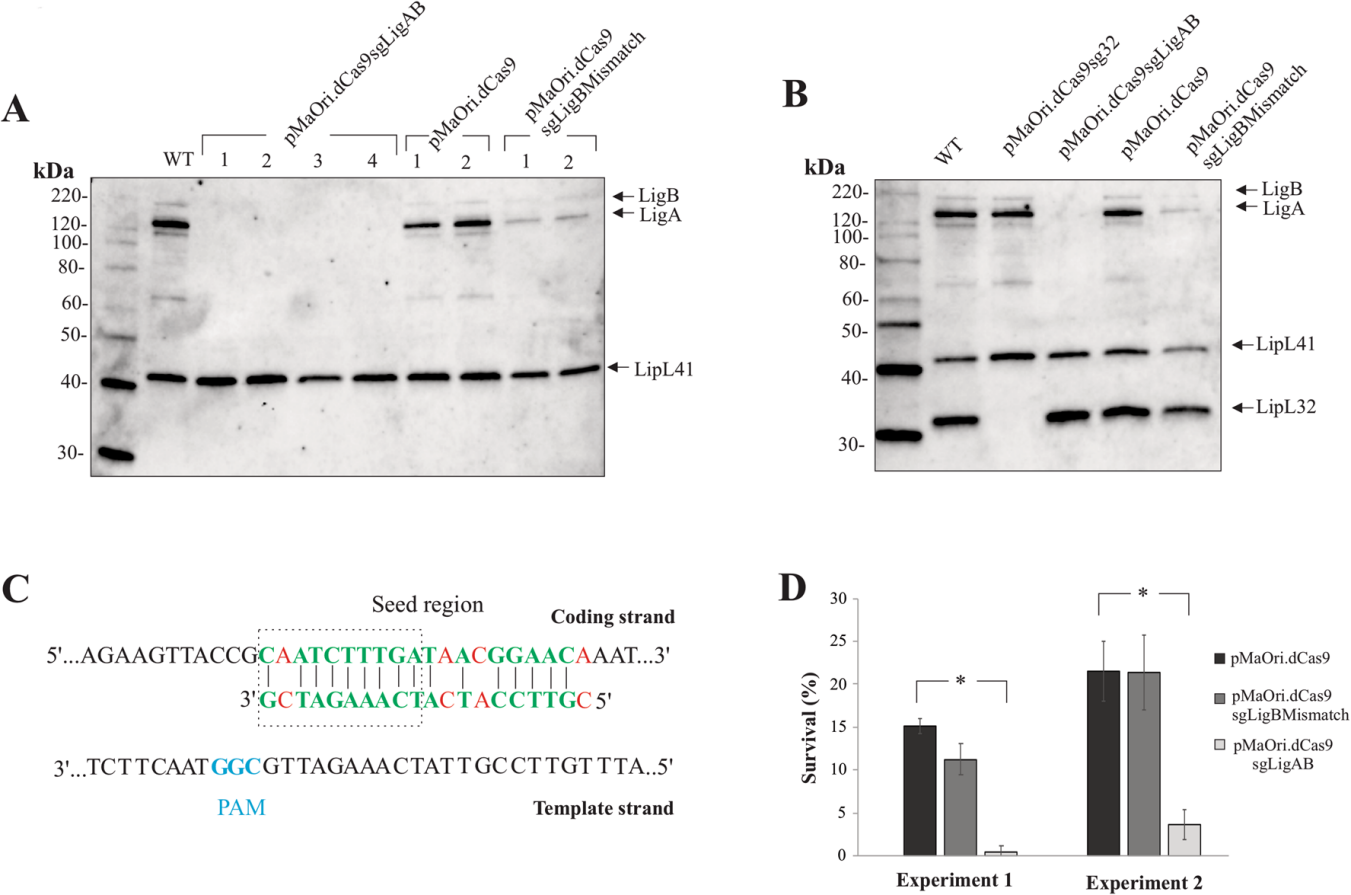
Concomitant LigA and LigB gene silencing reduces leptospiral serum resistance. (**A**) *L. interrogans* cells were transformed with plasmids pMaOri.dCas9sgLigAB, pMaOri.dCas9sgLigBMismatchControl or pMaOri.dCas9 alone, different colonies were picked, grown in liquid HAN plus spectinomycin and cellular extracts were evaluated by immunoblotting with anti-LigAB and anti-LipL41. (**B**) Representative extracts of each mutant obtained in this work were evaluated by immunoblotting with co-incubation with anti-LipL32, anti-LipL41 and anti-LigA/B. (**C**) Pairing of sgRNA expressed by pMaOri.dCas9sgLigBMismatchControl to *L. interrogans ligB* coding strand. Matches are presented in green and mismatches in red. Protospacer adjacent motif (PAM) NGG is highlighted in blue. Recombinant cells with different levels of LigA and LigB proteins were challenged with bovine serum (25%) for 2, 4 and 24 h and then plated in duplicate onto HAN plates. Colonies were counted and numbers were compared by Student's t-test. For each group, the number of cells recovered after incubation with heat-inactivated (HI) serum were considered as 100% survival.

Our results thus far highlight the application of CRISPRi to specifically silence a gene highly conserved amongst pathogenic species of leptospires. To further validate the use of CRISPRi as a universal tool to target any gene of choice, the concomitant silencing of both LigA and LigB in *L. interrogans* was performed by designing a sgRNA capable of pairing to a conserved region in the coding strand for both *ligA* and *ligB*, Table 1. By applying the optimized conjugation protocol as described above, an average of 25 colonies per plate, after 8 days, were obtained when *L. interrogans* was transformed with pMaOri.dCas9 and pMaOri.dCas9sgLigAB. Transconjugants were confirmed by PCR (data not shown). Selected colonies were grown in liquid HAN media and evaluated for the expression of LigA and LigB by immunoblotting with anti-LigA/B antibodies. When both dCas9 and specific sgRNA for *ligA* and *ligB* of *L*. *interrogans* were expressed, no LigA nor LigB protein could be detected in whole cell extracts, Fig. 5A,B.

Both LigA and LigB of *L*. *interrogans* have been extensively characterized. In contrast to *L*. *interrogans*, the genome of the pathogenic species *L*. *borgpetersenii* contains *ligB*, but not *ligA*. An sgRNA designed specifically for *ligB* of *L*. *borgpetersenii*, contained in pMaOri.dCas9sgLigBMismatch and which differs from that of the sgRNA specific for *ligB* of *L*. *interrogans* by 4 bp, was used as an additional control, Fig. 5C. In contrast to cells transformed with pMaOri.dCas9sgLigAB which showed no expression of LigA and LigB, reduced levels of expression were detected in cells transformed with pMaOri.dCas9sgLigBMismatch. We hypothesize that this reduced expression effect is due to the relaxed specificity for the target nucleotide sequence of *ligA* and *ligB* by pMaOri.dCas9sgLigBMismatch, Fig. 5C. Finally, sgRNA specific for *lipL32* had no effect on the expression of LigA or LigB, and conversely, sgRNA specific for *ligA* and *ligB* had no effect on the expression of LipL32, Fig. 5B.

*L. interrogans* cells silenced for LigA and LigB, or containing the control plasmids, were incubated with bovine serum for 24 h and cells were plated in HAN solid media for enumeration of viable leptospires. The bactericidal effect of bovine serum complement was initially confirmed in saprophytic *L. biflexa* after 2 h incubation, Supplementary Figure S4. *L. interrogans* recombinant cells containing plasmid pMaOri.dCas9 displayed an average of 18.3% survival rate after serum exposure (15.1 and 21.5% in experiment 1 and 2, respectively), followed by 16.3% survival when cells expressed reduced levels of LigA and LigB due to pMaOri.dCas9sgLigBMismatch (11.2 and 21.3%). Interestingly, when both proteins were completely silenced, only an average of 2% of cells could be recovered (*p* < 0.05, 0.5 and 3.6%), Fig. 5D, indicating a functional role for these proteins in resistance to serum, and as displayed by pathogenic species of *Leptospira*.

### *lipL32* gene silencing in a non-*interrogans* pathogenic *Leptospira*

**Figure 6:**
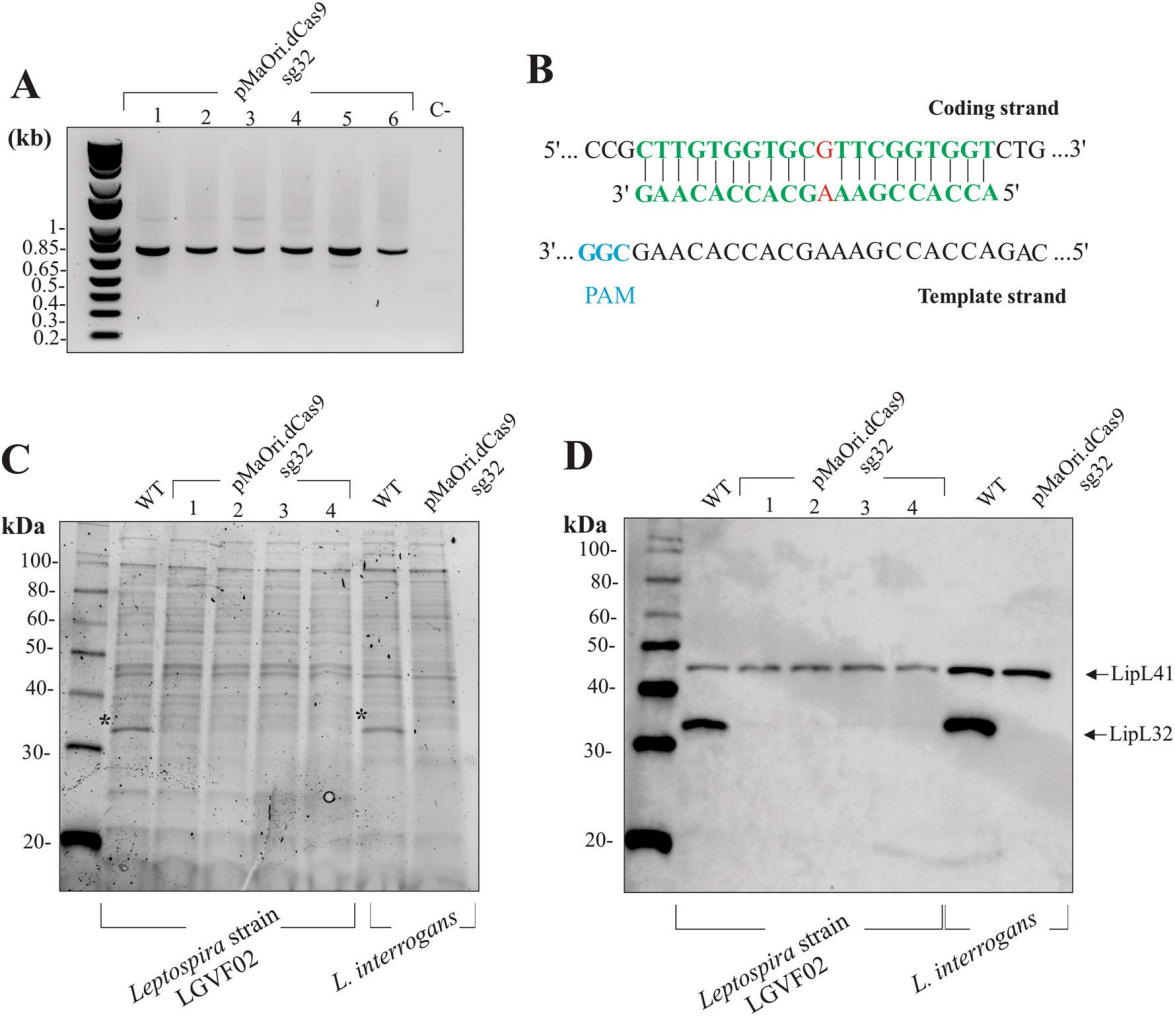
Reduction of LipL32 expression in an environmental non-*interrogans* species. A recently isolated pathogenic non-*interrogans Leptospira* strain LGVF02 was transformed with the plasmid pMaOri.dCas9sg32 and recovered colonies were evaluated by PCR with pMaOri2 primers (**A**). Based on the *lipL32* gene sequence, a mismatch is expected (**B**). Whole cell extracts from wild type (WT) and 4 transconjugants containing the plasmid were evaluated by SDS-PAGE (**C**) and immunoblotting (**D**) with anti-LipL32 and anti-LipL41 antibodies. Wild type or plasmid-containing *L. interrogans* were included as control.

To assess the feasibility of CRISPRi in alternative pathogenic species of *Leptospira*, a recent, non-*interrogans*, pathogenic isolate of *Leptospira* was employed. This isolate was recently recovered from soil samples in Puerto Rico and contains the pathogen specific gene *lipL32* (Nathan Stone et al., Northern Arizona University, manuscript in preparation). The plasmid pMaOri.dCas9sg32 was delivered by conjugation and after 6 days incubation, an average of 30 colonies were recovered on plates, which were further confirmed as true transconjugants by PCR with pMaOri2 primers (Fig. 6A). Based on the *lipL32* gene sequencing of the recent soil isolate (Nathan Stone et al. personal communication), a single mismatch could be observed between the sgRNA and the coding strand of the gene, Fig. 6B, with a conserved PAM. Based on the findings of mismatch tolerance of CRISPRi, an effect upon LipL32 protein expression is expected. Accordingly, protein extract analysis indicates that a major band of 32 kDa (*) could only be identified in wild type strain, Fig. 6C. Immunoblotting demonstrated that LipL32 protein levels were drastically reduced in the plasmid-containing cells compared to wild-type, though slight expression of LipL32 could still be detected when overexposed, Fig. 6D. Wild type and pMaOri.dCas9sg32-containing *L. interrogans* were included as controls for complete gene silencing.

## Discussion

Initially, genetic tools are developed using saprophytic species of *Leptospira*, due to their higher competence for exogenous DNA delivery and shorter colony formation time on agar plates; if the tool is proven efficient, it is next applied to pathogenic strains. The dawn of genetic manipulation in *Leptospira* spp. began with the development of an *E. coli-Leptospira* shuttle vector with DNA derived from temperate phage LE1, capable of replicating as a circular plasmid in the prophage state^34^. This vector contained a kanamycin resistance marker that could be delivered to the saprophyte *L. biflexa* by electroporation. Accordingly, the next breakthrough in the toolbox for genetic manipulation was gene replacement in *L. biflexa*, where Picardeau et al.^35^ applied a suicide vector containing the *L. biflexa flaB* gene disrupted by a kanamycin marker and selection of mutants with double cross-over event. In subsequent years, additional tools and techniques included the development of an RP4 derivative plasmid and demonstration of conjugative transfer between *E. coli* and *Leptospira* spp.^36^, construction of a transposon library in *L. interrogans* by mariner-based transposon Himar1^7^ and the development of a plasmid replicative in pathogenic *Leptospira* spp.^37^.

Until very recently, targeted gene disruption in species of *Leptospira* relied on homologous recombination; however, the requirement of double cross-over events drastically reduces the frequency of knockout mutants recovered, especially for pathogenic strains^36^. In lieu of this, the use of an episomally-delivered gene silencing system has emerged as an alternative strategy. In this scenario, the only event that needs to occur is plasmid delivery, and thus, efficient mutant recovery. The first attempt of gene knockdown in species of *Leptospira* was carried out by Pappas and Picardeau^38^ by employing the *Xanthomonas* transcription activator-like effector (TALE). Even though the authors demonstrated the potential role of LigA and LigB proteins during infection, and validated TALE as a feasible tool, the technique requires the synthesis of large and specific genes for each target, making it costly and laborious^39^.

The prokaryote immunity type II CRISPR/Cas system is a robust, specific and affordable biotechnological tool for manipulating the genome of several organisms. Since Cas9-induced cleavage of *Leptospira* genome is lethal to the bacteria, a variant of Cas9, dCas9 (“dead” Cas9) with a point mutation within the nuclease domains^6,18^ has been employed to achieve gene silencing rather than disruption, in a strategy called CRISPR interference (CRISPRi). CRISPRi was successfully applied in the saprophyte *L. biflexa* by the construction and delivery by electroporation of a plasmid capable of expressing both dCas9 (pMaOri.dCas9) and sgRNA^16^.

Genetic manipulation in pathogenic strains is inherently difficult, due to their reduced transformability in comparison to saprophytic leptospires^36^, and longer colony formation time in EMJH solid media taking from 4–6 weeks to appear^40^, with plates becoming more susceptible to dehydration and contamination. More recently, a new growth media for pathogenic leptospires, HAN, has been reported, facilitating growth not just as the traditionally used growth temperature of 29 °C, but also at 37 °C^23^. Prior to transconjugant recovery described here, HAN was modified for optimal growth of pathogenic leptospires as colonies on agar media to include 0.4% rabbit sera and incubation at 37 °C with 3% CO_2_: this effectively reduced colony formation time of *L. interrogans* serovar Copenhageni strain FIOCRUZ L1-130 to 1 week.

To demonstrate the applicability of CRISPRi, we selected the highly transcribed gene *lipL32*, which encodes the major outer membrane protein LipL32 and highly conserved amongst pathogenic *Leptospira* species. Application of the optimized conjugation protocols rendered rapid and efficient transconjugant recovery, which were silenced for LipL32 expression, as demonstrated by LipL32, RT qPCR, proteomics and immunoblot. Additionally, a stable phenotype was preserved even when no antibiotic selective pressure was applied to the cells in vitro. Conjugation parameters were optimized for *L. interrogans* serovar Copenhageni strain Fiocruz L1-130; however, given the large number of diverse species and serovars of pathogenic leptospires that have been identified to date, optimal conjugation parameters should be determined empirically for alternative strains.

In order to demonstrate the universal applicability of CRISPRi to pathogenic leptospires, alternative gene targets were assessed. Given their extensive characterization, as well as conserved gene sequence between them, sgRNA were designed to target both *ligA* and *ligB*. When leptospires were transformed with plasmids pMaOri.dCas9sgLigAB, which contains a sgRNA targeting the conserved region of both genes, neither LigA nor LigB proteins could be observed in recovered transconjugants, in contrast to the expression observed in either *L. interrogans* wild type cells or those containing pMaOri.dCas9 alone. This is the first time where both LigA and LigB proteins were completely silenced in pathogenic leptospires.

Surprisingly, when *L. interrogans* cells were transformed with plasmids containing a mismatch control sgRNA, designed for *ligB* of *L. borgpetersenii*, a slight reduction in LigA and LigB protein expression levels was observed; alignment between the protospacer 20 bp sequence and the *ligA* and *ligB* DNA sequence of *L. interrogans* identified 4 bp mismatches, and juxtaposed to an NGG PAM. We hypothesize that this accounts for the partial gene silencing due to a decreased binding frequency and strength of the RNA:DNA heteroduplex. It has been previously shown that mismatches between guide RNA and target DNA can attenuate, or even abolish, dCas9-mediated repression^17,41–43^. In addition, Cress et al*.*^43^ indicated that a single mismatch in the PAM-proximal seed region is not sufficient to abolish dCas9-mediated repression. Partial gene silencing is interesting in the context of studies for genes considered essential and though nucleotide mismatches can account for incomplete silencing, the outcome can be unpredictable. When partial gene silencing is desired, a specific sgRNA can be designed to perform Watson and Crick base pairing with the template strand of a gene, as previously performed to demonstrate the essential function of the chaperone DnaK in *L. biflexa*^16^.

In previous work, when TALE was applied to silence genes encoding LigA and LigB, these proteins could still be detected in the cellular extracts of recombinant leptospires, indicating that protein expression was reduced, but not abolished^38^. In this work, the application of CRISPRi targeting either LipL32 or LigA and LigB proteins prevented any detection of expression of these proteins, demonstrating complete gene silencing, and corroborating the effectiveness of this new genetic tool. Pathogenic leptospires, in contrast to saprophytic species, are partially resistant to the bactericidal effect of the complement system^44–46^. Several well-characterized proteins of *Leptospira* have been shown to function by recruiting host complement proteins and regulators^47–51^ as a strategy to provide resistance against host serum. The heterologous expression of LigA and LigB proteins enhanced the survival of *L. biflexa* in human serum compared to wild type strain^52^. In this sense, CRISPRi-mediated LigA and LigB silencing in pathogenic *L. interrogans* drastically reduced bacterial survival upon exposure to bovine serum, confirming the role of these proteins in resistance to serum, and thus accounting for the virulence attenuation observed by Pappas and Picardeau^38^ when Lig proteins levels were reduced.

Previous mutagenesis studies in pathogenic *Leptospira* were limited to different serovars within the species *L. interrogans*^7,38,40,53,54^. In this work, we demonstrate for the first time the genetic manipulation of a pathogenic non-*interrogans* species, (Nathan Stone, Northern Arizona University, manuscript in preparation). This isolate was recently isolated from soil samples and is awaiting further characterization, but nevertheless demonstrates the applicability of CRISPRi to other pathogenic species of *Leptospira*.

The demonstration of complete, stable and specific gene silencing, combined with the ease in obtaining constructs concordant with the relatively rapid recovery of mutants in modified HAN solid media is a significant milestone in leptospirosis research that will facilitate novel approaches to elucidate pathogenic mechanisms of infection.

## Supplementary Information

Supplementary Information.
