# Supplementary material for: Genetic manipulation of pathogenic Leptospira: CRISPR interference (CRISPRi)-mediated gene silencing and rapid mutant recovery at 37 °C
Source: Sci Rep. 2021 Jan 19;11:1768. doi: 10.1038/s41598-021-81400-7 (PMC7815788; doi:10.1038/s41598-021-81400-7)
Supplement: Supplementary file 1 — Supplementary Information. [file 41598_2021_81400_MOESM1_ESM.pdf]

Supplementary figures for:

Genetic manipulation of pathogenic *Leptospira*: CRISPR interference (CRISPRi)-mediated gene silencing and rapid mutant recovery at 37ºC

Fernandes, L.G.V.[1,2,*], Hornsby, R.L.[1], Nascimento, ALTO[2,#], Nally, J. E.[1,#]

[1]Infectious Bacterial Diseases Research Unit, National Animal Disease Center, Agricultural Research Service, United States Department of Agriculture, Ames, IA, U.S.A.

[2]Laboratório de Desenvolvimento de Vacinas, Instituto Butantan, São Paulo 05503-900, Brazil.

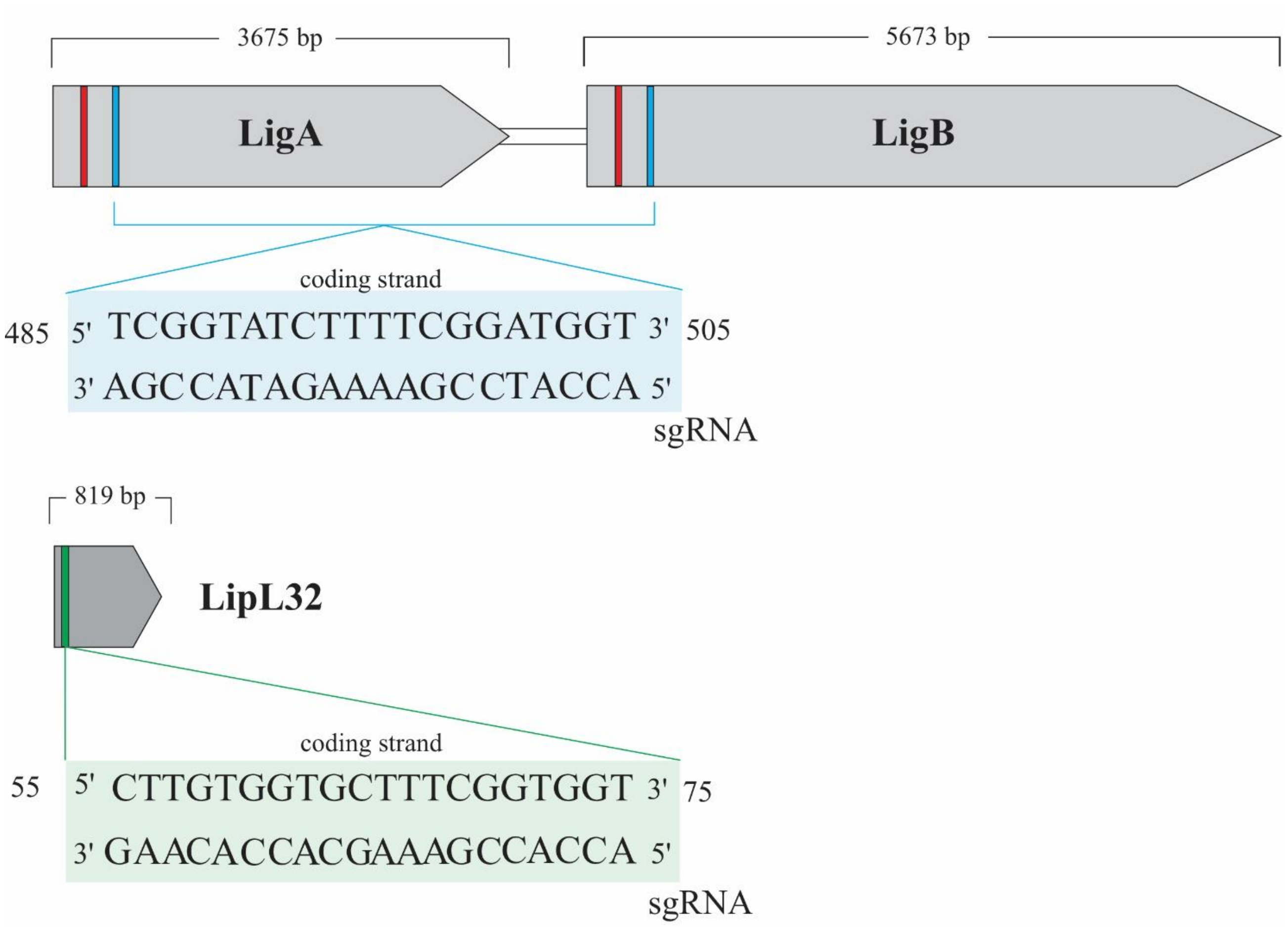

Supplementary Figure 1. Representation of sgRNA targets. *ligA*, *ligB* and *lipL32* gene were selected for targeted silencing. sgRNA were designed to contain a 20bp 5’ sequence capable of pairing to the coding strand of the conserved region of both *ligA* and *ligB* (blue) and *lipL32* (green). An sgRNA designed for *L. borgpetersenii ligB* gene was included as a mismatch control to both *ligA* and *ligB* of *L. interrogans* (red) and base pairing is depicted in Figure 5C. The scaffold regions of the sgRNA are not represented.

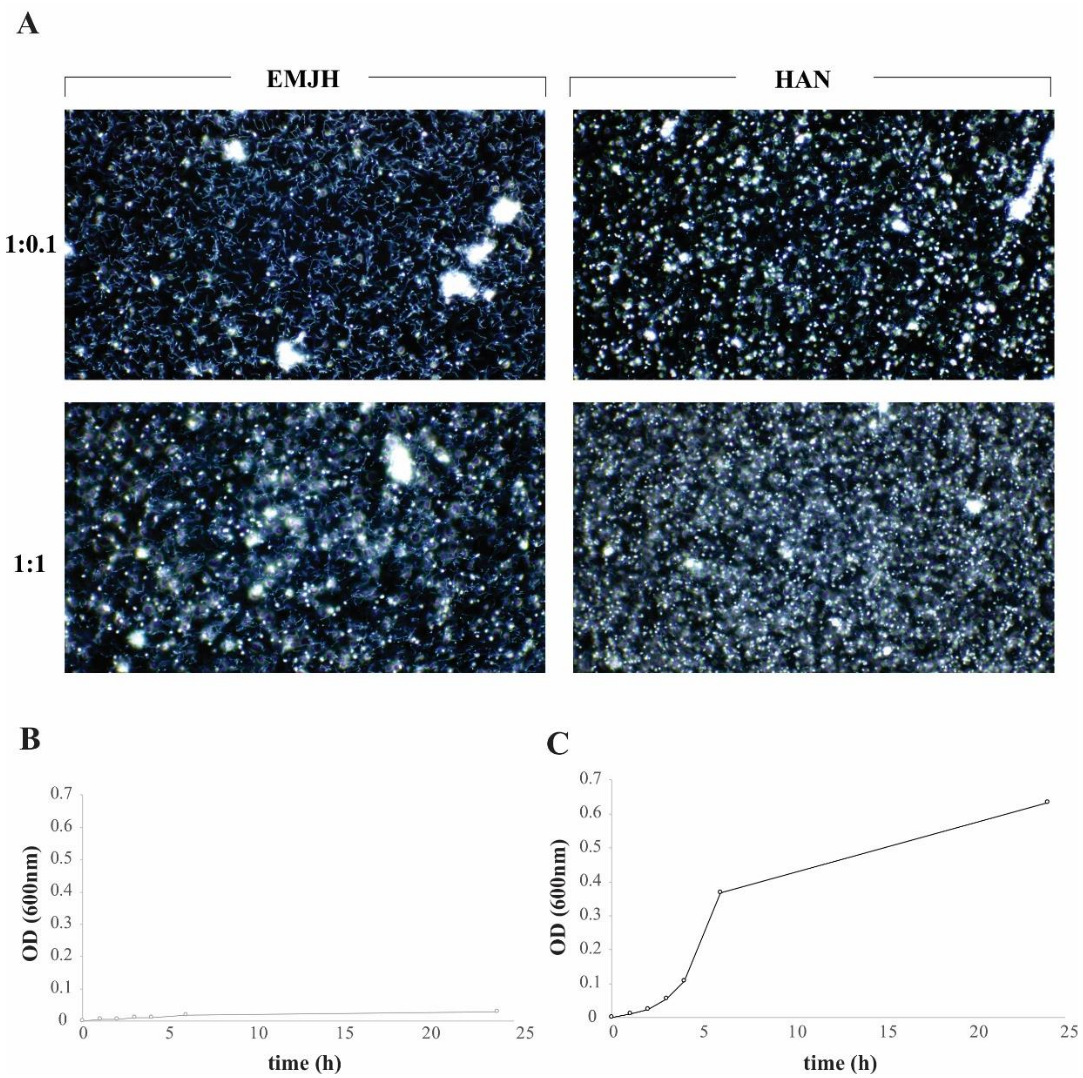

Supplementary Figure 2. Dark-field microscopy of *Leptospira : E. coli* conjugation. Bacteria after 24h filter mating at different media and proportions were collected and visualized under dark field microscope **(A)**. The growth of wild type *E. coli β2163* was evaluated in both EMJH and HAN media **(B)**.

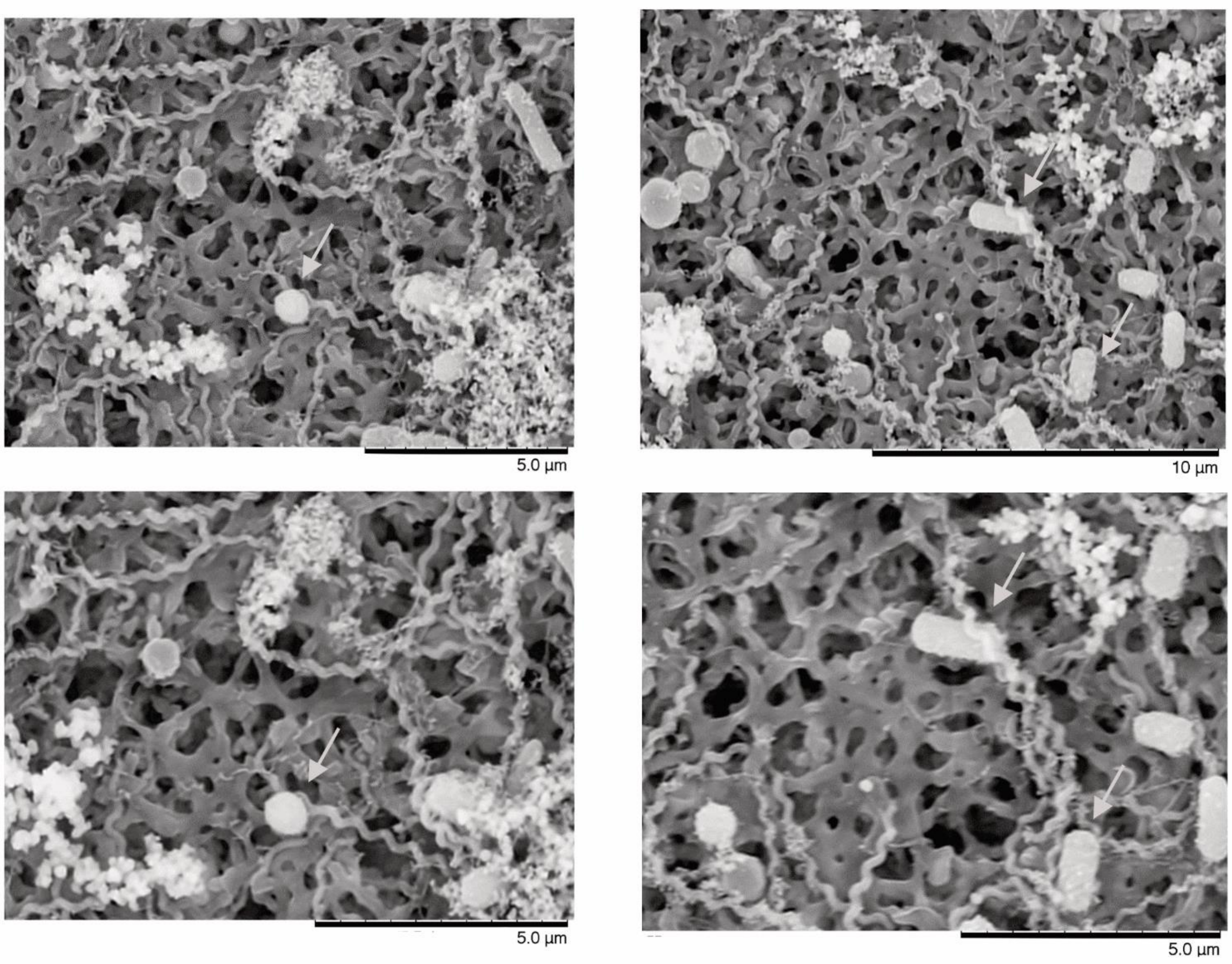

Supplementary Figure 3. Scanning electron microscopy of conjugation. Conjugation reactions were recovered from filters, diluted and prepared for electron microscopy. Arrows indicate the close association of donor *E. coli* to recipient *L. interrogans*.

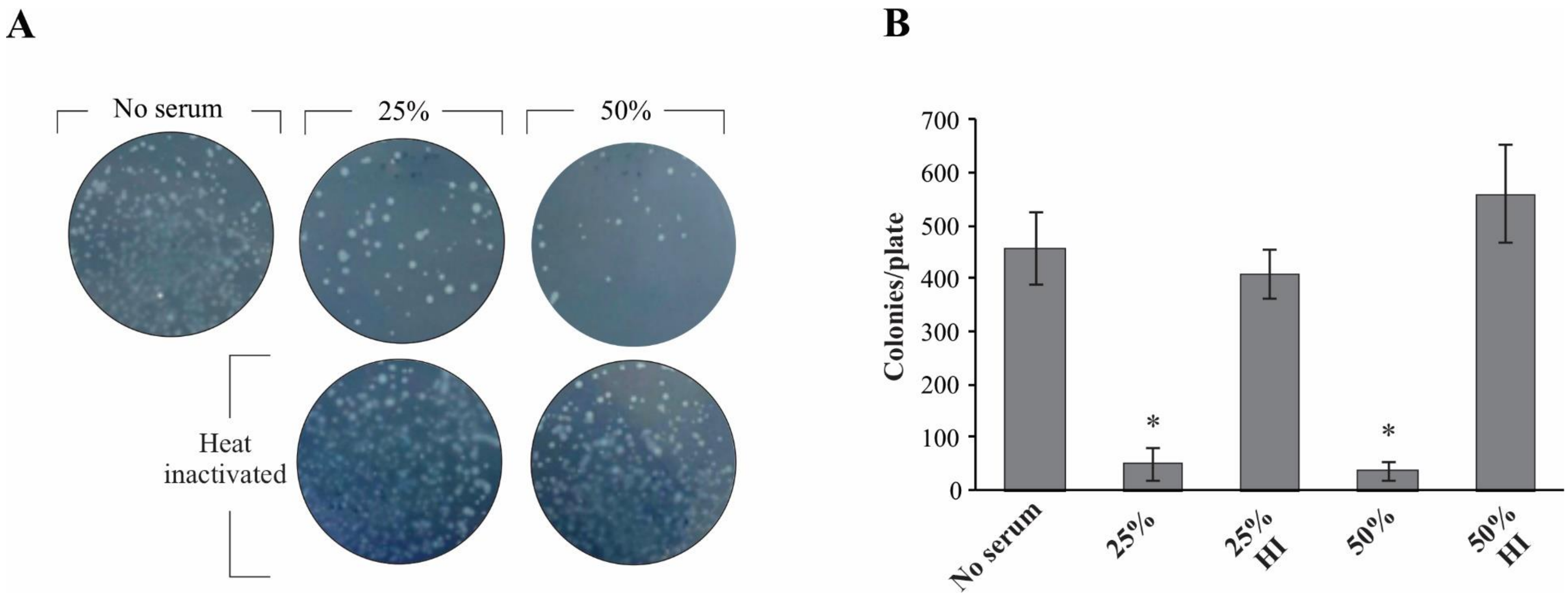

Supplementary Figure 4. Bactericidal effect of bovine serum upon *L. biflexa*. Saprophytic *L. biflexa* cells were incubated with 25 or 50% bovine serum, heat inactivated (HI) or not, for 2h at 29ºC and then plated onto EMJH agar plates **(A)** and then counted **(B)**. SE bars represent standard errors, and * denotes significance of p-value < 0.05.
